# Replacing the Langmuir Isotherm with the Statistical Thermodynamic Fluctuation Theory

**DOI:** 10.1021/acs.jpclett.4c00281

**Published:** 2024-03-27

**Authors:** Seishi Shimizu, Nobuyuki Matubayasi

**Affiliations:** † York Structural Biology Laboratory, Department of Chemistry, University of York, Heslington, York YO10 5DD, United Kingdom; ‡ Division of Chemical Engineering, Graduate School of Engineering Science, Osaka University, Toyonaka, Osaka 560-8531, Japan

## Abstract

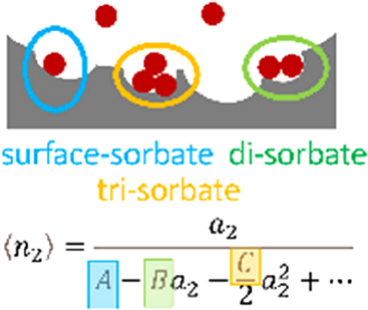

In the age of all-atom simulations, primitive isotherm models, such as Langmuir, BET, and GAB, are still used widely for analyzing experimental data. However, their routine applications to complex materials are not in line with their underlying assumptions (i.e., statistically independent adsorption sites with no interfacial structural changes), which manifests as the temperature dependence of the monolayer capacity. Our proposal is to replace these models with the statistical thermodynamic fluctuation theory because the ABC isotherm derived from it (i) contains these primitive models as its special cases, (ii) is applicable to any interfacial geometry, and (iii) is linked to molecular distribution functions, sharing the same language as simulations. Rectifying the inability of the primitive isotherm models to handle attractive and repulsive interactions consistently leads to a reconsideration of how physical interpretations should be attributed to the isotherms of empirical origin (e.g., Freundlich).

Our goal is to fill the ever-widening gulf between atomistic simulations and classical isotherm models. The most common isotherms, still used to this day for analyzing experimental data, assume

(a) statistically independent site-specific (“localized”) binding on a monolayer of uniform surfaces (the Langmuir model)^1^ and
(b) (a) plus site-specific layer-by-layer adsorption (the BET and GAB models)^2^

that hardly resemble the systems to which these models are applied, such as powders, pores, food, and construction materials.^2−4^ Nevertheless, successful fittings have been demonstrated routinely,^5,6^ suggesting that the applicability of these primitive models may be much wider than their original assumptions.

To liberate the Langmuir, BET, and GAB from their restrictive assumptions, the early attempts aimed to incorporate “mobile” adsorption mechanisms by combining the Gibbs adsorption isotherm with the hypothetical equations of states (EOS) for the interfacial “phase”.^3,7^ However, the simplest EOS (the ideal gas and van der Waals) did not lead to Langmuir, BET, and GAB but to the Volmer^8^ and Hill–de Boer^9,10^ models. In fact, the EOS underlying the Langmuir, BET, and GAB models turned out to be complex.^7^ Until recently, the dichotomy of the “localized” and “mobile” adsorption mechanisms persisted.^11^

We have shown recently that Langmuir, BET, and GAB models are special cases of the isotherm from the statistical thermodynamic fluctuation theory (called the ABC isotherm).^11−13^ Its key parameters, representing mono-, di-, and trisorbate interactions at the interface, are universal and model-independent.^11−13^ Its foundation, the Kirkwood–Buff integrals^14,15^ and their generalization to interfaces^16^ can capture both localized and mobile adsorptions, while providing a link between isotherms and molecular distribution functions of sorbates, which is a natural language for simulation.^11^ Powders and pores no longer need to be force-adapted to uniform, site-specific, layer-by-layer adsorption assumed by the BET model, which has made the surface area estimation procedure more straightforward.^13^

The objectives of this Perspective are

I. To replace the oversimplified Langmuir model with the ABC isotherm.
II. To replace the classical approach for treating surface heterogeneity (i.e., “to describe the energetic heterogeneity by assuming that the adsorbent surface consists of a collection of locally homogeneous surfaces” obeying the Langmuir model) with the statistical thermodynamic fluctuation theory.^17^

Objective I will be achieved by demonstrating that

- the ABC isotherm, without any restrictions on surface geometry, uniformity, and site-specificity, contains the Langmuir model as its special case.

In contrast, the previous approach, based on the Langmuir model, suffers from

- the violation of its core assumption, the temperature independence of “monolayer capacity” as a logical consequence of the statistical independence of binding sites, which has been well-documented; and
- the inability to treat attractive and repulsive interactions on an equal footing, arising from the assumption that “adsorbate–adsorbate interactions on the surface are neglected”.^18^

To achieve Objective II, we take the Freundlich model as an illustrative example because its physical meaning, despite its widespread use, needs to be established. We will demonstrate that Objective II will be achieved by circumventing the above interpretation of surface heterogeneity and noting that

- the statistical thermodynamic fluctuation theory provides a simple derivation of the Freundlich model and reveals sorbate–sorbate repulsion as its underlying mechanism.

In contrast, according to the classical view, the “Freundlich isotherm model expression defines the heterogeneity of the surface as well as the exponential distribution of the active sites and the active sites energies.”^19^ However, its theoretical foundation, the adsorption energy distribution based on locally-Langmuir isotherms, suffers from

- the violation of its core assumption, the temperature independence of adsorption energy distribution function, as the consequence of the statistical independence of the locally homogeneous (Langmuir) surfaces; and
- the inability to capture sorbate–sorbate interactions arising from the limitations of the site-specific binding assumption.

Through these steps, statistical thermodynamic fluctuation theory will identify and overcome the core limitation of the traditional site-specific isotherm models, i.e., the inability to treat attraction and repulsion on the same theoretical grounds.

## Statistical Thermodynamic Fluctuation Theory

Here we outline the fluctuation sorption theory whose fundamental principles are

i. the generalized Gibbs isotherm, defined directly via statistical ensembles;^16^
ii. the geometry-free dividing surface, applicable to arbitrary interfacial geometry or porosity;^16^
iii. the excess number relationship, linking the gradient of an isotherm to sorbate–sorbate excess number.^12,16^

Through these principles, the fluctuation sorption theory can quantify, from the isotherm’s shape alone, the sorbate–sorbate and sorbate–interface interactions underlying an isotherm.^12,13,16^

**Figure 1:**
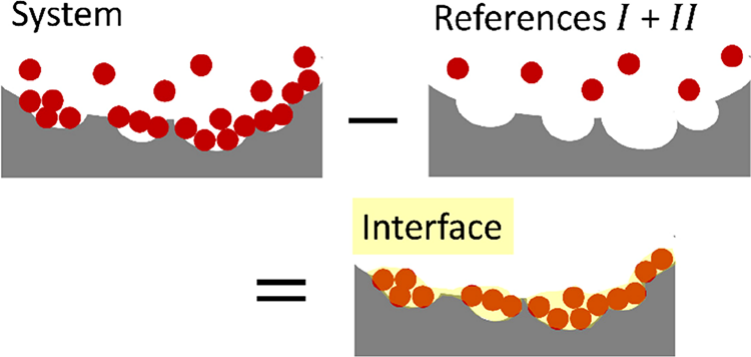
A schematic representation of the interface as the difference between the system and the reference systems, with sorbates (red circles), sorbent (gray), and the interfacial volume *v* (yellow highlighted region) illustrated intuitively for adsorption. Note that the boundary of the interface (i.e., the farthest end of the yellow region) is defined by the convergence of the sorbate–surface distribution function (see main text).

This Perspective aims for a clear illustration of principles i–iii by focusing on adsorption for simplicity, even though our theory is applicable to adsorption and absorption alike, and in the presence of sorbent structural changes.^12,13^ The “interactions” in this theory are the sorbate number correlations at the interface,^14,20−23^ as a generalization of the Kirkwood–Buff solution theory.^15,16^ The surface–sorbate interaction is quantified by the surface–sorbate surface excess,^12,13,16^
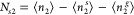
1aas the difference in ensemble-averaged (⟨ ⟩) sorbate numbers between the system with an interface (⟨*n*_2_⟩) and the solid (⟨*n*_2_^*s*^⟩) and vapor (⟨*n*_2_^*g*^⟩) reference systems^13,16^ (Figure 1; see Appendix A for notation). From the excess number, the sorbate–surface Kirkwood–Buff integral (KBI), *G*_*s*2_, is defined as^12,13,16^
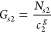
1bwhere *c*_2_^*g*^ = ⟨*n*_2_^*g*^⟩/*v* is the sorbate concentration in the vapor phase (where *v* is the volume of the interface) which depends on sorbate activity and the temperature.

Our isotherm theory, in the most general form, has been founded on surface excesses (see refs (13) and (16)). However, introducing the “interface” explicitly is beneficial for comparison with simulations and classical models (Figure 1). This can be implemented by the two postulates. First, the interface is finite ranged;^13,16^ hence, *N*_*s*2_ and *G*_*s*2_ converges within a finite distance from the interface. Consequently, *G*_*s*2_ can be evaluated via eq 1a solely by the number difference within the finite volume *v* of the interface. In the case of adsorption, *G*_*s*2_ can be evaluated using
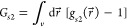
2awhich, in principle, is accessible to simulation directly via the surface–sorbate distribution function, , defined as the concentration of the sorbate at the position r⃗, normalized by the concentration of the vapor reference system. This is an advantage of adopting *G*_*s*2_ as the foundation for sorption isotherms. Note that ⟨*n*_2_⟩, ⟨*n*_2_^*g*^⟩, and ⟨*n*_2_^*s*^⟩ are the numbers of particles in volume *v* of the interface, vapor (gas) reference, and solid reference, respectively, following the introduction of *v*. Second, since sorbates in vapor and solid reference systems are dilute, ⟨*n*_2_^*s*^⟩ and ⟨*n*_2_^*g*^⟩ can be neglected in comparison to the amount of sorbates at the interface (⟨*n*_2_⟩),^13^ such that
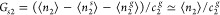
2b

These two postulates facilitate the quantification of the sorbate–sorbate interaction at the interface via the sorbate–sorbate KBI, defined as^12,13,16^
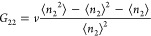
3which has a direct link to the sorbate–sorbate distribution functions, *g*_22_, via^12,13,16^
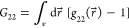
4where  is the distribution of sorbate pairs separated by r⃗ within the interface normalized by taking  at the  limit. Alternatively, sorbate–sorbate excess number, *N*_22_,
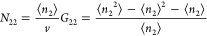
5not only provides an intuitive interpretation of sorbate–sorbate interaction as the excess number of sorbates around a probe sorbate but also a useful expression for the gradient of an isotherm.^12,13,16^ Thus, the explicit introduction of “interface” and *v*(13) enables a direct comparison of our theory with simulations and classical isotherm models.

The isotherm is a plot of ⟨*n*_2_⟩ against the activity of sorbate , where  is the concentration of the saturated vapor. According to the following fundamental relationships of the fluctuation sorption theory, *N*_22_ and *G*_22_ are related to the gradient of an isotherm, via^12,13,16^
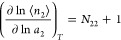
6and
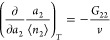
7Equations 6 and 7 not only reveal the underlying sorbate–sorbate interaction directly from an experimental isotherm but also serve as the *isotherm-generating relationships*;^24^ a systematic method of deriving isotherm equations. We emphasize here that eqs 6 and 7 are the simplified versions of our general theory applicable to adsorption and absorption alike (see eqs 2a and 3 of ref (13)).

**Figure 2:**
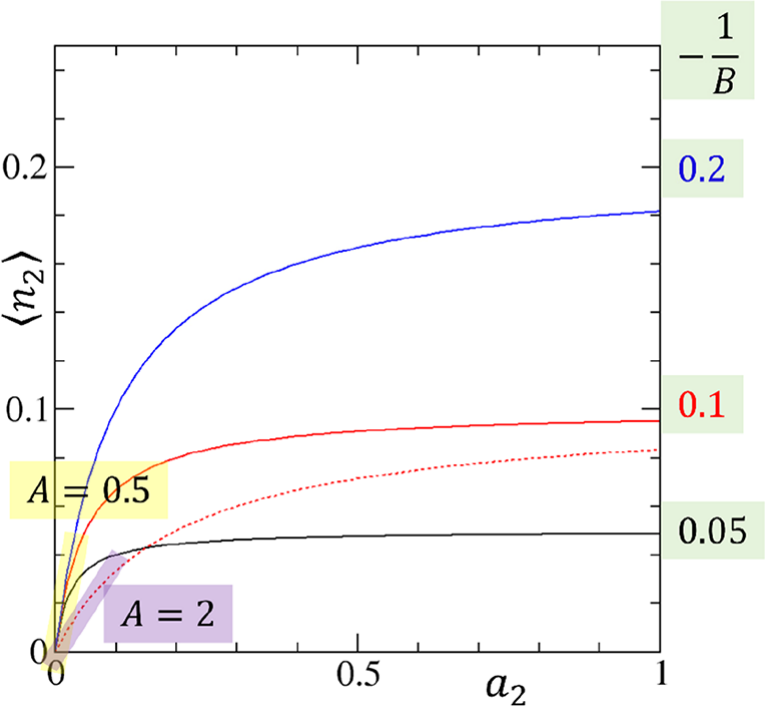
An intuitive guide to the physical meaning of the parameters *A* and *B* of the ABC isotherm with *C* = 0. 1/*A* governs the initial gradient via ⟨*n*_2_⟩ ≃ *A*^–1^*a*_2_ with *A* = 0.5 (solid lines highlighted with yellow) and *A* = 2 (dotted red line, highlighted with purple). −1/*B* governs the saturating capacity with −1/*B* = 0.05 (black), 0.1 (red), and 0.2 (blue); red dotted line (*A* = 2, −1/*B* = 0.1).

Let us demonstrate how an isotherm equation can be derived directly from the isotherm-generating relationship (eq 6 or 7).^24^ To do so, let us express *G*_22_/*v* in terms of the disorbate (*B*) and trisorbate (*C*) interactions via
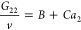
8Integrating eqs 7 and 8 yields
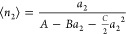
9where, via a comparison of eq 9 with eq 2b, the integration constant *A* can be linked to the sorbate-interface KBI via^11,12^
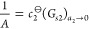
10where  is the saturated vapor concentration. We emphasize here that the isotherm (i.e., ⟨*n*_2_⟩ as a function of *a*_2_) can be derived by integrating its gradient (eq 7); in doing so, the parameter *A*, introduced as the integration constant, plays an important role, providing a direct link (via eq 10) to the sorbate-interface KBI. The physical meaning of the parameters *A* and *B* for adsorption are illustrated in Figure 2 (and the most general form of the ABC isotherm, applicable to adsorption and absorption alike, are found in eqs 4–9 of ref (13)). The above derivation did not assume site-specific binding onto statistically independent binding sites on a uniform interface. Equation 9 is called the ABC isotherm; when *C* = 0, it is called the AB isotherm. As is clear from its functional shape, the ABC isotherm (eq 9) contains the Langmuir, BET, and GAB models as its special cases (see ref (13)). The uniform, layer-by-layer, site-specific adsorption mechanism underlying the Langmuir, BET, and GAB has been replaced by sorbate–surface, disorbate, and trisorbate interactions.^13^ Its ramification to specific surface area evaluation is detailed in refs (13) and (11).

## Replacing the Langmuir Model

The Langmuir model has been used successfully for more than a century.^18^ Hence, the case for replacing it with the more general ABC isotherm must be made with an unambiguous criterion for Langmuir’s breakdown. By “breakdown” we do not mean poor fitting. Even under successful fitting, the underlying mechanism breaks the Langmuir model assumptions,^25^ as we will demonstrate below.

Let us start with the statistical thermodynamic rederivation that led to the clarification of the mechanism underlying the Langmuir model:^1^ (i) “only one adsorbed molecule can be attached”^1^ to a binding site, (ii) the binding sites are uniform, and (iii) “[t]he adsorbed states belonging to any one surface atom are assumed to be independent of whether surrounding surface atoms are holding adsorbed molecules or not”.^1^ Based on (i)–(iii), adsorption on an interface, composed of *n*_*m*_ statistically independent adsorption sites (known as the monolayer capacity) with single maximum occupancy, each with the binding constant *K*_*L*_, can be expressed via the Langmuir model:^1^
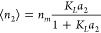
11aor, when the sorbate pressure (*P*_2_) is chosen as the variable instead of *a*_2_,^26,27^
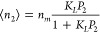
11bHere, “[t]he number of adsorption sites does not change with the system temperature for a physically and chemically inert adsorbent”,^25^ which is in line with the requirement that *n*_*m*_ (i.e., the number of subsystems within a macroscopic interface) must be independent of the temperature. However, the violation of this requirement has been well documented,^25^ as will be discussed later.

Our proposal is to replace the Langmuir model with the ABC isotherm, thereby eliminating the oversimplified restrictions (i)–(iii) in the previous paragraph. This can be achieved by redeploying *n*_*m*_ and *K*_*L*_ as the parameters for the ABC isotherm via the following correspondence (of eq 9 to eqs 11a and 11b):^11,12^
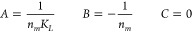
12This correspondence applies both to the *a*_2_ and *P*_2_ representations of the Langmuir model; for the *P*_2_ representation (eq 11b),  replaces eq 10 while the correspondence of *B* to KBI remains unchanged (eq 8 with *C* = 0).

In contrast, for the Langmuir model (and its multisite extensions), the breakdown of the temperature independence of the monolayer capacity has been well documented (see the in-depth review by Sircar^25^); more examples are found in the applications of the Langmuir model to pressure swing adsorption (PSA) and temperature swing adsorption (TSA) for gas separation, purification, and capture.^26,27^ In one such example, the adsorption of CO_2_ on activated carbon (Table 1),^28^ the temperature independence criterion of *n*_*m*_ has not been met. When “the basic assumptions of the Langmuir model”^25^ are broken, the first strategy to overcome such a problem is to replace the Langmuir model with the ABC isotherm via eq 12 (Table 2), reinterpreting the isotherm via the mono-, di-, and trisorbate interactions at the interface. (For an alternative approach based on eq 6, see Appendix B.) We emphasize that even if sorbate exclusion is determined predominantly by the site-specific mechanism as assumed by the Langmuir model,^1,25^ the ABC isotherm can handle it naturally; in this case, *B* = −1/*n*_*m*_ (eq 12), simply becomes temperature independent. Unlike the Langmuir model, there is no restriction on how *A*, *B*, and *C* should depend on the temperature for the ABC isotherm.

**Table 1:** AB Isotherm Parameters Determined from the Langmuir Fitting Not in Line with the Temperature Independence of the Monolayer Capacity for the CO_2_ Adsorption on Activated Carbon^a^^,^^b^

*T*/°C	*n*_*m*_/mmol g^–1^	*K*_*L*_/MPa^–1^	*A*^–1^/mmol g^–1^ MPa^–1^	*B*/mg mol^–1^
25	10.83	1.142	12.3	–0.0923
45	10.33	0.771	7.96	–0.0968
65	9.21	0.603	5.55	–0.109
100	8.00	0.387	3.10	–0.125
140	6.88	0.256	1.76	–0.145

a *n*_*m*_ and *K*_*L*_ reported by Schell et al.^28^

b The units for *B*, mg mol^–1^, come from m^3^ mol^–1^ for *G*_22_ and m^3^ mg^–1^ for *v*.

**Table 2:** Expressions for *N*_22_ and *G*_22_/*v* for the AB, Langmuir, and Freundlich Isotherms

	AB	Langmuir	Freundlich
*N*_22_	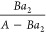	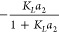	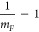
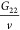	*B*	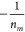	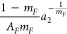

The simplicity of the above resolution contrasts with the traditional approach, for which violation of the criterion meant a need for lateral interactions between sorbates.^25^ However, such a view is based on inconsistent treatment of attractive and repulsive interactions. This can be demonstrated by the following reinterpretation of *n*_*m*_ via eqs 8 and 12:^11,12^
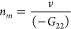
13

According to the KB theory, −*G*_22_ signifies the covolume (i.e., the volume territory per sorbate). Consequently, eq 13 (i.e., interfacial volume *v* per covolume −*G*_22_) signifies the number of sorbates at the interface.^11,12^ This marks a departure from the traditional view: the “monolayer capacity” reflects the repulsive interaction between sorbates (*G*_22_ < 0). In fact, the common view that “adsorbate–adsorbate interactions on the surface are neglected”^18^ in the Langmuir model fails to consider repulsions as “interactions”.

Indeed, treating attractive and repulsive interactions on an equal footing is essential for a systematic elucidation of Types I–III isotherms (see ref (11)). However, inconsistent treatment of attraction and repulsion has been the source of historical confusion, among which the most notable are biomolecular solvation and solubilization.^22,29^

Historically, the need for incorporating attractive interactions in an *ad hoc* manner led to the plethora of isotherm models, each assuming a different mechanism (e.g., as summarized in Table 2 of Sircar^25^). This has made it difficult to identify the “correct” mechanism solely from the goodness of fit.^6^ Instead, the ABC isotherm (containing up to trisorbate interactions) is the simplest case of a universal approach for incorporating multiple-body sorbate interactions successively. This gives systematic strategies when the ABC isotherm is not sufficient via (i) incorporating multiple-body interactions beyond trisorbate and (ii) capturing the heterogeneity of the interface (see the next paragraph).

## Replacing the Local-Langmuir Approach for Treating Interfacial Heterogeneity and the Freundlich Model

Our second objective (Objective II) is to replace the classical approach for treating surface heterogeneity by adopting the Langmuir model for “a collection of locally homogeneous surfaces”.^17^ As an illustrative example, we re-examine the important consensus that a fit to the Freundlich model signifies surface heterogeneity.^19^ Note that the Freundlich model,
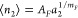
14with the parameters *A*_*F*_ and *m*_*F*_ (>1) was proposed originally as an empirical relationship. However, from our theory, the physical meaning of the Freundlich model is clear, as can be demonstrated by its straightforward derivation from the following characteristic relationship for the excess number,^24^
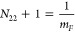
15Combining eq 15 with the *N*_22_ representation of the isotherm-generating relationship (eq 6), one arrives at the Freundlich model (eq 14). This derivation identifies the sorbate–sorbate interaction underlying the Freundlich model, via
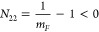
16The constant negative excess number in eq 16 signifies sorbate–sorbate exclusion. However, we have shown with eqs 8, 9, and 13 that the AB isotherm (and the Langmuir model as its special case) also represents sorbate–sorbate exclusion. What is the difference between the two? The underlying mechanisms can be summarized (Table 2) as

- a constant negative *N*_22_ for the Freundlich model;
- a constant negative *G*_22_/*v* for the AB isotherm and the Langmuir model.

The two different expressions for the constancy of sorbate exclusions arise from the dual expressions (eqs 6 and 7, via *N*_22_ and *G*_22_/*v*) for the isotherm-generating relationship from the fluctuation theory. (For an alternative approach to Table 2, see Appendix B.)

In contrast to the clarity of the statistical thermodynamic fluctuation theory, the classical approach based on local-Langmuir isotherms is complicated and contradictory. To demonstrate this, let us examine the foundation of the classical approach closely via statistical thermodynamics. The basic assumption is that the isotherm, ⟨*n*_2_⟩, is a collection of local Langmuir isotherms, θ(ε,*a*_2_), via^30^
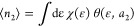
17where ε is the adsorption energy, related to the Langmuir constant *K*_*L*_, via^31−33^
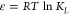
18and χ(ε) is the adsorption energy distribution function (AEDF). In this approach, ε is assumed to be independent of the temperature. This is consistent with the identification of ε to the isosteric heat of adsorption for the (single term) Langmuir model (eq 11a).

To examine the validity of local Langmuir decomposition (eq 17), let us start with a macroscopic interface consisting of statistically independent patches.^34,35^ Let the patch type be denoted by τ, each with *N*^(τ)^ patches in total. Because of statistical independence, the partition function is multiplicative; hence the amount of sorption is patchwise-additive, as^34,35^
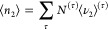
19where  represents the mean sorbate number within a patch of type τ. Because ε does not depend on the temperature, it can be adopted to designate a patch. Using a discrete series ({ε_τ_} = ε_1_, ε_2_, ...), eq 19 can be rewritten as
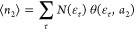
20When the total number of patches in the interface is finite, *N*(ε_τ_) is a macroscopic quantity and is independent of the temperature. Under this condition,  is an ensemble average. Note that eq 20 becomes the multiple-term ABC isotherm by adopting the ABC isotherm for θ(ε_τ_, *a*_2_), which is the replacement of the multisite-Langmuir model (Langmuir’s Case II).^18,30^

Increasing the number of divisions in eq 20, the discrete relationship (eq 20) becomes the continuous one (eq 17) with the introduction of AEDF. As a generalization of *N*(ε_τ_) in eq 20 and *n*_*m*_ in eq 11a, χ(ε) is considered to be temperature-independent. However, the temperature independence of χ(ε) is not fulfilled for the Freundlich model. Note that an analytical evaluation of χ(ε), due to its limiting properties (*a*_2_ → 0),^31−33,36,37^ has been circumvented via the Sips model^32^ (whose limiting form is the Freundlich model) and by adding Henry’s law term.^38^ Both approaches (see refs (17) and (38) and p 102, eq 4.5.19, of Everett and Rudzinski^33^) led to χ(ε) containing , which depends on the temperature. Thus, AEDF underlying the surface heterogeneity interpretation of the Freundlich model is in contradiction to the required temperature independence of χ(ε).

To summarize, the fluctuation sorption theory can replace the local-Langmuir (AEDF) approach for the interpretation of the isotherms with more “complex” origins, such as Freundlich (Objective II).

## Concluding Remarks

The statistical thermodynamic fluctuation theory serves as a common language between atomistic simulations and isotherm equations for fitting experimental data. With this theory, adsorption isotherms can be interpreted via the sorbate distribution at the interface, quantified via molecular distribution functions and their integrations (the interfacial Kirkwood–Buff (KB) integrals).^11^ The ABC isotherm, which captures the sorbate–surface, disorbate, and trisorbate interactions at the interface, contains the Langmuir, BET, and GAB isotherms as its special cases.^11−13^ Because of the versatility of the KB integral, the ABC isotherm can be applied to “localized” and “mobile” adsorptions alike, even to the systems with absorption and sorbent structural changes.^11^ The ABC isotherm has also been generalized to sorption from solution.^39^

In contrast, the Langmuir model is incapable of treating repulsive interactions in the same way as the attractive. As a result, a change of sorbate–sorbate repulsion with temperature is in violation of its foundation, i.e., the statistical independence of binding sites.^25^ The ABC isotherm, in contrast, can capture the change of sorbate–sorbate interaction (attractive and repulsive) merely as a change of *G*_22_, via the values of *B* and *C*.^11−13^

The unequal treatment of attraction and repulsion brought further complications when the Langmuir model was adopted as the local isotherm. Expressing the Freundlich model via the superposition of local Langmuir isotherms was achieved^17^ yet in contradiction with the criterion of temperature independence of AEDF. The fluctuation theory, in contrast, provides a clear interpretation of the Freundlich model as the manifestation of sorbate–sorbate repulsion.

Thus, the fluctuation sorption theory is applicable across the classical categories of sorbate behavior, referred to as the “patchwise” versus “random” surface topologies and the “mobile” versus “localized” adsorbates.^33^ This achievement has practical ramifications: recycling historic fitting parameters from the conventional models to reveal the underlying adsorption mechanism in a language common to atomistic simulations.

## Appendix A. Terminology and Notation

We use the term “sorption” unless there is a need to distinguish adsorption, because the same fundamental relationships (eqs 6 and 7) apply both to absorption or desorption.^12^ Throughout this paper, we adopt the statistical thermodynamic notation consistent not only with our papers on sorption^12,16^ but also with solvation.^29^ The sorbent and sorbate are referred to as species 1 and 2, and *n*_2_ and *a*_2_ are the number and activity of sorbates, respectively.^12,16^ The ensemble average is denoted by ⟨ ⟩, through which the sorption isotherm is the dependence of ⟨*n*_2_⟩ on *a*_2_ at the temperature *T*. *R* is the gas constant. The statistical thermodynamic notation corresponds with the IUPAC notation via *n* = ⟨*n*_2_⟩ and *a*_2_ = *p*/*p*_0_.

## Appendix B. Application of the Excess Number Relationship

Here, we demonstrate that the fluctuation theory based on eq 6 can attribute a clear physical meaning to the Sips model, which gives a better fit to the CO_2_ adsorption on activated carbon.^28^ The Sips model can be expressed via the empirical parameters *m*, *P*_*m*_, and , as

B1

where the range of *m* is restricted as 0 < *m* < 1 and *a*_2_ is proportional to *P*_2_. Combining eq B1 with eq 6 yields

B2

which is consistent with our result for our cooperative isotherm.^34,35^ Since 0 < *m* < 1, *N*_22_ < 0, which signifies sorbate–sorbate exclusion, consistent with our reinterpretation of the Langmuir model via the ABC isotherm. Thus, our theory provides two complementary approaches for sorbate–sorbate interactions via (i) *G*_22_/*v* and (ii) *N*_22_.
